# Unilateral pulmonary edema after minimally-invasive redo-double valve replacement procedure—case report

**DOI:** 10.3389/fcvm.2025.1662660

**Published:** 2025-10-23

**Authors:** Dan Wang, Jia Yang, Haibo Ren

**Affiliations:** Department of Critical Care Medicine, Wuhan Asian Heart Hospital, Wuhan, China

**Keywords:** re-expansion pulmonary edema, veno-venous extracorporeal membrane oxygenation, extracorporeal membrane oxygenation, cardiopulmonary bypass, minimally-invasive cardiac surgery

## Abstract

This case report describes a patient with a history of mechanical aortic and mitral valve replacements who developed prosthetic valve stenosis years later and underwent a minimally invasive, thoracoscopic-assisted double valve re-replacement. Postoperatively, the patient developed a rare but life-threatening re-expansion pulmonary edema (RPE), which led to severe acute respiratory distress syndrome (ARDS), with rapid deterioration into refractory hypoxemia. Despite comprehensive conventional supportive management—including lung-protective ventilation, diuresis, anti-inflammatory therapy, and other measures—adequate oxygenation could not be maintained. Venovenous extracorporeal membrane oxygenation (VV-ECMO) was emergently initiated. With ECMO support, the patient's lungs were able to rest and recover. After 10 days of support, ECMO was successfully discontinued, and the patient eventually recovered and was discharged. This case aims to explore the pathophysiological mechanisms of RPE, management strategies for complications following minimally invasive cardiac surgery, and to emphasize the critical role of VV-ECMO in treating refractory respiratory failure, as well as the importance of multidisciplinary team (MDT) collaboration.

## Introduction

Prosthetic valve dysfunction (e.g., stenosis or thrombosis) is a potential serious long-term complication following cardiac valve replacement. Repeat sternotomy is associated with significant trauma and high surgical risk. Minimally invasive cardiac surgery (MICS) has become an important option for reoperation due to advantages such as reduced trauma and faster recovery. However, MICS also carries the risk of common major surgical complications, among which ARDS is one of the most severe. RPE is a rare form of unilateral acute lung injury triggered by rapid re-expansion of a collapsed lung, with a high fatality rate. When conventional therapy fails, VV-ECMO can serve as a ultimate salvage modality to maintain oxygenation and buy time for lung recovery (1–3).

## Case history

The patient was a 54-year-old male diagnosed with rheumatic valvular heart disease, severe mitral stenosis, and moderate aortic stenosis with moderate aortic insufficiency. In 2002, the patient underwent mechanical mitral and aortic valve replacement under general anesthesia, hypothermia, and CPB. Over the past six years, the patient experienced worsening dyspnea during moderate-to-vigorous physical activity. Transthoracic echocardiography revealed post-operative mechanical valve dysfunction, with pannus formation on the valve frame and associated valve stenosis, peak velocity of the aortic valve: 5.0 m/s, pressure difference: 100 mmHg, LVEF56%. His electrocardiograph (ECG) indicated atrial fibrillation, and the pulmonary function test indicated mild restrictive ventilatory dysfunction. The patient's vital signs and laboratory test results were within normal parameters. The patient and his family members strongly desired to avoid the huge trauma caused by traditional open-chest surgery and requested to undergo minimally invasive surgery instead. Consequently, minimally invasive mechanical aortic and mitral valve replacements were carried out.

Following anesthetic induction with a left double-lumen endotracheal tube, the surgical procedure was performed through the right third anterolateral intercostal space. Single lung ventilation (left side) was employed. CPB was established via the right femoral artery and vein after heparin administration. A thoracoscope was inserted through the fifth intercostal space at the anterior axillary line. Through the third intercostal minimally invasive incision, after sternum dissociation, the adhered pericardium was accessed, exposing the ascending aorta, left atrium, and right atrium. An additional cannula for left atrial drainage was introduced through the right superior pulmonary vein. Lung ventilation was suspended, and constant supportive pressure was set to 4 cmH_2_O. Following direct aortic cross-clamping (ACC), antegrade cardioplegic solutions were perfused to induce cardiac arrest. The thoracic cavity was filled with carbon dioxide throughout the procedure. The CPB procedure lasted 400 min during the surgery, with an ACC time of 286 min.

**Figure 1:**
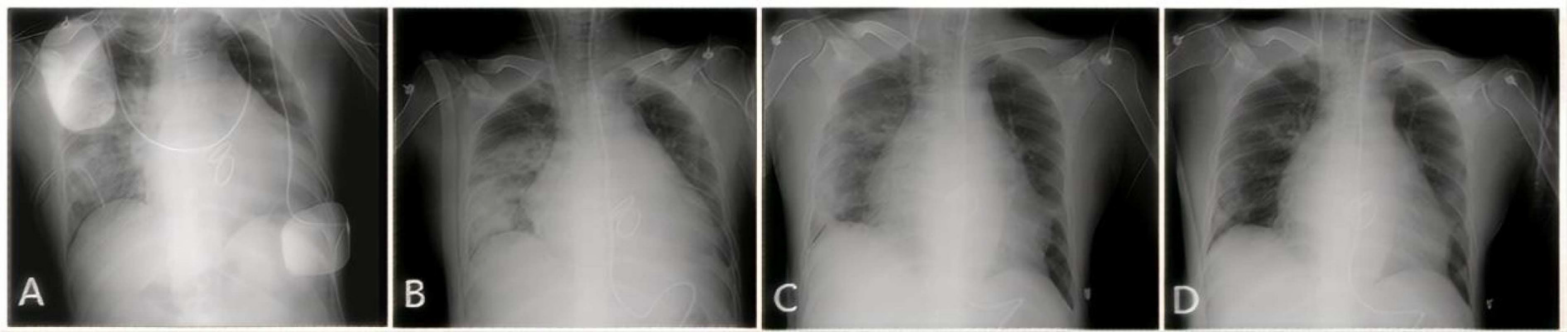
CXR showing the resolution of RPE. **(A)** Day 1: Extensive opacification throughout the right lung. **(B)** Day 3: Initial improvement in aeration. **(C)** Day 10: Marked clearing of the opacification. **(D)** Day 30 (pre-discharge): Nearly complete resolution.

Upon arrival at the intensive care unit (ICU), the patient's oxygen saturation fluctuated between 70% and 90% despite escalation to 100% of inspired oxygen. Arterial blood gas analysis revealed a PaO_2_ of 59 mmHg. The patient experienced a continuous decline in the oxygenation index (PaO_2_/FiO_2_) to <100 mmHg. Large volume of serous secretions were aspirated through the endotracheal tube. The CXR (Figure 1A) revealed partial atelectasis of the left lung and diffuse infiltrates in the right lung, which confirmed the presence of RPE in the right lung. A lung-protective ventilation strategy was immediately instituted, with a target tidal volume of 6 ml/kg, a driving pressure of 16 cmH_2_O, and a positive end-expiratory pressure (PEEP) of 12 cmH_2_O. Subsequently, a lung-protective ventilation strategy — with a target tidal volume of 6 ml/kg, driving pressure of 16 cmH_2_O, and positive end-expiratory pressure (PEEP) of 12 cmH_2_O — was implemented, along with sedation, muscle relaxation, and diuretic therapy for pulmonary edema. However, the patient's oxygenation failed to improve, and severe respiratory acidosis developed in conjunction with extremely unstable vital signs. The primary postoperative manifestation was refractory hypoxemia. Transthoracic echocardiography was limited by pulmonary edema and air artifacts, precluding a full assessment. However, the limited views available indicated that cardiac function was satisfactory. Following a rapid discussion among a multidisciplinary team comprising cardiothoracic surgery, intensive care, and the ECMO team, the decision was made to initiate VV-ECMO support. A 19Fr cannula was percutaneously inserted into the right internal jugular vein. Additionally, a 23Fr venous cannula was introduced through the common femoral vein to the inferior vena cava and positioned at the right atrium orifice (Figure 3A). VV-ECMO circuit was established via the right internal jugular and right femoral veins at a flow rate of 4.0 L/min with 100% oxygen. Following ECMO initiation, the patient's oxygenation improved rapidly, allowing for a significant reduction in mechanical ventilation parameters to achieve a “lung rest” strategy (FiO_2_ < 40%, tidal volume 4–6 ml/kg, PEEP 10–12 cmH₂O).

**Figure 2:**
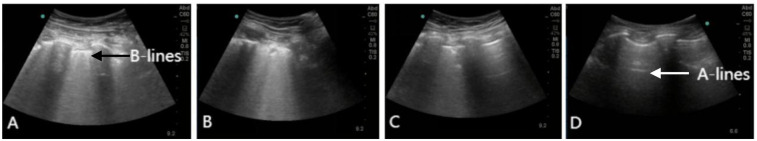
Lung ultrasound showing the resolution of RPE. **(A)** Lung ultrasound on day 1 demonstrating diffuse B-lines in the right lower lung. **(B–D)** Serial lung ultrasounds performed on day 3, 10, and 30 show progressive improvement. Real-time lung ultrasound identified A-lines (white arrow) and B-lines (black arrow).

**Figure 3:**
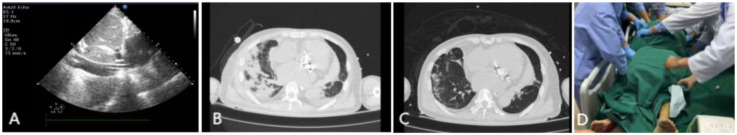
Imaging findings during VV-ECMO therapy. **(A)** Ultrasound confirming the positioning of the vena cava catheter tip at the orifice of the right atrium. **(B)** Thoracic CT on postoperative day 6 demonstrating pulmonary edema in the right lung. **(C)** Follow-up CT on postoperative day 13 showing significant improvement in right lung exudation. **(D)** Illustration of VV-ECMO therapy combined with PPV.

During his ICU stay, serial assessments—including CXR (Figures 1A–D), lung ultrasounds (Figures 2A–D), computed tomography (CT; Figures 3B,C), and blood gas analyses—were obtained to monitor his condition. The chest CT revealed significant exudation in the right dorsal segment. Based on this finding, prone positioning ventilation (PPV) was initiated as an adjunct to the ongoing VV-ECMO therapy to treat the RPE (Figure 3D). By postoperative day 10, the patient's CXR demonstrated marked improvement and lung auscultation findings were unremarkable, enabling the discontinuation of VV-ECMO. The patient was successfully extubated on postoperative day 12 and was subsequently discharged on postoperative day 30 without sequelae. Transthoracic echocardiography prior to discharge demonstrated normal function of the prosthetic valve. Informed consent was obtained from the patient for the publication of his clinical details and images.

## Discussion

Although the mortality and stroke risks of MICS are comparable to those of traditional surgery, its cardiopulmonary bypass (CPB) and aortic cross-clamp times are typically longer (4). Prolonged procedure time and lung collapse due to one-lung ventilation are common predisposing factors for life-threatening complications such as RPE after cardiac surgery, particularly in MICS, which to some extent increases the risk of postoperative complications. The incidence of unilateral pulmonary edema after MICS ranges from 1.6% to 25% (5, 6). The clinical manifestations of RPE vary widely, from being asymptomatic with only radiological abnormalities to acute respiratory failure with life-threatening severe hypoxemia. Therefore, close postoperative monitoring of pulmonary imaging and oxygenation status is crucial for the early recognition and management of RPE. Although its exact pathogenesis and risk factors are not fully elucidated, its occurrence is associated with multiple factors. Preoperative factors primarily include the patient's existing comorbidities and physiological status, such as underlying diseases like pulmonary hypertension (especially mean pulmonary arterial pressure >35 mmHg), chronic obstructive pulmonary disease, and diabetes; preoperative use of steroids or immunosuppressants; and elevated levels of inflammatory markers such as C-reactive protein. These factors may increase pulmonary vascular permeability and susceptibility to inflammatory responses, laying the pathological foundation for RPE. Intraoperative factors play a key role in the development of RPE. MICS often requires one-lung ventilation, leading to prolonged collapse of the non-ventilated lung and causing regional ischemia. Upon reperfusion, massive generation and release of reactive oxygen species and various inflammatory mediators significantly increase pulmonary capillary permeability, leading to edema (7). In this case, the elevation of the patient's inflammatory indicators after the operation supports the mechanisms of ischemia-reperfusion and inflammation. Furthermore, prolonged CPB and aortic cross-clamping can exacerbate systemic inflammatory responses, further promoting lung injury (8). Other postoperative risk factors include massive transfusion of blood products.

Although chest x-ray is convenient and provides a preliminary assessment of postoperative cardiopulmonary status, its accuracy in identifying imaging findings and underlying pathology is limited due to its anteroposterior projection and the constraints of the ICU environment. Lung ultrasound can serve as a powerful alternative to chest x-ray; compared to CT or x-ray, it allows more accurate classification of lung morphology. The anterior chest wall region is most accessible and useful for lung morphological classification (9). An increased number of B-lines on ultrasound is associated with elevated serum N-terminal pro-brain natriuretic peptide levels and an increased incidence of major adverse cardiovascular events (10). Due to its non-invasiveness, bedside availability, and widespread accessibility in various healthcare settings, lung ultrasound has an irreplaceable value in the diagnosis and management of RPE (11). It is also crucial for disease diagnosis, monitoring of disease progression, and evaluation of treatment efficacy. Bedside lung ultrasound facilitates rapid early recognition and management of RPE; real-time monitoring of cardiopulmonary function and early clinical intervention are also vital, enabling timely diagnosis and treatment.

For severe or life-threatening RPE, initiation of extracorporeal life support is often required immediately after surgery. When conventional supportive therapy is ineffective, emergent initiation of VV-ECMO has become an effective rescue strategy to reverse refractory hypoxemia and prevent the occurrence of multiple organ dysfunction syndrome. VV-ECMO provides adequate gas exchange, improves systemic oxygenation and promotes carbon dioxide removal, while also helping to reduce the risk of ventilator-induced lung injury (12). VV-ECMO has shown favorable clinical outcomes in treating severe ARDS, and its early application can significantly improve patient survival (13). Current evidence indicates that combining prone positioning ventilation (PPV) with VV-ECMO is safe and feasible (14). Multiple studies have demonstrated that early combination of ECMO and PPV in patients with severe ARDS can further reduce mortality and shorten ECMO support duration. First, PPV promotes redistribution of gas within the lungs by altering body position, reduces collapse in dorsal lung regions, improves ventilation/perfusion (V/Q) matching, and thereby significantly increases the oxygenation index (PaO_2_/FiO_2_), more effectively correcting refractory hypoxemia. Second, postural changes in PPV help reduce compression from the heart and mediastinal structures on dorsal lung tissue, promoting alveolar recruitment, particularly in gravity-dependent areas. Finally, postural changes in PPV facilitate drainage of airway secretions, reduce the risk of atelectasis, improve lung mechanics, and aid in controlling pulmonary infection and reducing inflammation. In summary, the combination of PPV during VV-ECMO is a highly valuable lung-protective and recruitment strategy, particularly suitable for ARDS patients in whom conventional mechanical ventilation fails to maintain oxygenation or who have severe pulmonary inhomogeneity, as it provides life support while creating favorable conditions for recovery from the primary disease.

The successful management of this case benefited from the following factors: First, the rapid recognition of a rare but serious complication. Given the rapid progression of RPE, timely diagnosis based on clinical presentation and imaging features was the first step towards initiating correct treatment. Second, after maximal conventional therapy (including lung-protective ventilation and PPV) failed, the multidisciplinary team (MDT)—including cardiac surgery, ICU, and ECMO teams—responded swiftly and decisively, initiating VV-ECMO in a timely manner, which bought precious time for recovery of lung function and was critical to the successful rescue. ECMO broke the vicious cycle of “hypoxia-lung injury,” providing a decisive window for lung repair (15). With ECMO support, the ventilator could be set to an ultra-protective mode, implementing a “lung rest” strategy that effectively avoided further ventilator-induced lung injury and promoted healing of the lung parenchyma.

## Conclusion

For high-risk patients with a history of cardiac surgery, minimally invasive redo valve surgery is a feasible option, but vigilance against the risk of RPE leading to severe ARDS is essential. Once refractory respiratory failure difficult to correct with conventional therapy occurs, early consideration of VV-ECMO support should be given. A well-coordinated multidisciplinary team and proficient ECMO management skills are fundamental to the successful treatment of such critically ill patients. Timely recognition, continuous monitoring, and effective intervention are key elements in managing RPE. This case provides valuable insights and references for managing similar complex clinical scenarios.

## Data Availability

The original contributions presented in the study are included in the article/Supplementary Material, further inquiries can be directed to the corresponding author.

## References

[1] Lizwan M Lee WD Chong JJ Chua KC. Severe re-expansion pulmonary edema after minimally invasive mitral valve surgery: a case report and review of the literature. JTCVS Tech. (2023) 21:78–82. 10.1016/j.xjtc.2023.08.008 37854824 PMC10580166

[2] Prakasa SA Alatas A. Re-Expansion pulmonary edema following minimally invasive cardiac surgery: a case report. Ann Card Anaesth. (2024) 27(4):361–3. 10.4103/aca.aca_30_24 39365134 PMC11610773

[3] Hu Y Guo K Shi Y. Unilateral pulmonary edema after minimally-invasive bentall procedure—case report. Heliyon. (2024) 10(9):e29911. 10.1016/j.heliyon.2024.e29911 38707428 PMC11066307

[4] Puehler T Friedrich C Lutter G Kornhuber M Salem M Schoettler J Outcome of unilateral pulmonary edema after minimal-invasive mitral valve surgery: 10-year follow-up. J Clin Med. (2021) 10(11):2411. 10.3390/jcm10112411 34072399 PMC8198899

[5] Renner J Lorenzen U Borzikowsky C Schoeneich F Cremer J Haneya A Unilateral pulmonary oedema after minimally invasive mitral valve surgery: a single-centre experience. Eur J Cardiothorac Surg. (2018) 53(4):764–70. 10.1093/ejcts/ezx399 29186375

[6] Khalil NH Anders R Forner AF Gutberlet M Ender J. Radiological incidence of unilateral pulmonary edema after minimally invasive cardiac surgery. J Cardiothorac Vasc Anesth. (2020) 34(1):151–6. 10.1053/j.jvca.2019.07.006 31405722

[7] Fujita N Miyasaka K Okada O Katayama M Miyasaka K. Localized pulmonary edema in the middle and Inferior lobes of the right lung after one-lung ventilation for minimally invasive mitral valve surgery. J Cardiothorac Vasc Anesth. (2015) 29(4):1009–12. 10.1053/j.jvca.2014.04.006 25129646

[8] Kim HC Suh KH Lee YC. Severe bilateral Re-expansion pulmonary edema successfully managed with extracorporeal membrane oxygenation after robot-assisted mitral valve repair surgery. J Cardiothorac Vasc Anesth. (2016) 30(4):1038–41. 10.1053/j.jvca.2015.10.001 26776748

[9] Pierrakos C Smit MR Pisani L Paulus F Schultz MJ Constantin JM Lung ultrasound assessment of focal and non-focal lung morphology in patients with acute respiratory distress syndrome. Front Physiol. (2021) 12:730857. 10.3389/fphys.2021.730857 34594240 PMC8476947

[10] Platz E Campbell RT Claggett B Lewis EF Groarke JD Docherty KF Lung ultrasound in acute heart failure: prevalence of pulmonary congestion and short- and long-term outcomes. JACC Heart Fail. (2019) 7(10):849–58. 10.1016/j.jchf.2019.07.008 31582107 PMC8409324

[11] Smit MR Mayo PH Mongodi S. Lung ultrasound for diagnosis and management of ARDS. Intensive Care Med. (2024) 50(7):1143–5. 10.1007/s00134-024-07422-7 38656359

[12] Tonna JE Abrams D Brodie D Greenwood JC Rubio Mateo-Sidron JA Usman A Management of adult patients supported with venovenous extracorporeal membrane oxygenation (VV ECMO): guideline from the extracorporeal life support organization (ELSO). ASAIO J. (2021) 67(6):601–10. 10.1097/MAT.0000000000001432 33965970 PMC8315725

[13] Mi MY Matthay MA Morris AH. Extracorporeal membrane oxygenation for severe acute respiratory distress syndrome. N Engl J Med. (2018) 379(9):884–7. 10.1056/NEJMclde1804601 30157406

[14] Lyu G Cai T Jiang W Liu M Wang X. [Comparison of efficacy between veno-venous extracorporeal membrane oxygenation (VV-ECMO) and VV-ECMO combined with prone position ventilation for the treatment of acute respiratory distress syndrome]. Zhonghua Wei Zhong Bing Ji Jiu Yi Xue. (2021) 33(3):293–8. 10.3760/cma.j.cn121430-20200805-00563 33834969

[15] Bertini P Marabotti A Meani P Sangalli F Paternoster G. Rising above the limits of critical care ECMO: a narrative review. Medicina (Kaunas). (2025) 61(2):174. 10.3390/medicina61020174 40005292 PMC11857283

